# Cytoreductive Surgery with the PlasmaJet Improved Quality-of-Life for Advanced Stage Ovarian Cancer Patients

**DOI:** 10.3390/cancers15153947

**Published:** 2023-08-03

**Authors:** Gatske M. Nieuwenhuyzen-de Boer, Hanane Aamran, Caroline B. van den Berg, Sten Willemsen, Jurgen M. J. Piek, Nathalie Reesink-Peters, Marianne Maliepaard, Helena C. van Doorn, Suzanne Polinder, Heleen J. van Beekhuizen

**Affiliations:** 1 Department of Gynecologic Oncology, Erasmus MC Cancer Institute, University Medical Center Rotterdam, 3015 GD Rotterdam, The Netherlands; h.aamran@erasmusmc.nl (H.A.); c.vandenberg@erasmusmc.nl (C.B.v.d.B.); m.maliepaard@erasmusmc.nl (M.M.); h.vandoorn@erasmusmc.nl (H.C.v.D.); h.vanbeekhuizen@erasmusmc.nl (H.J.v.B.); 2 Department of Obstetrics and Gynecology, Albert Schweitzer Hospital, 3318 AT Dordrecht, The Netherlands; 3 Department of Epidemiology, Erasmus MC, 3015 GD Rotterdam, The Netherlands; s.willemsen@erasmusmc.nl; 4 Department of Biostatistics, Erasmus MC, 3015 GD Rotterdam, The Netherlands; 5 Department of Obstetrics and Gynecology, Catharina Cancer Institute, 5623 EJ Eindhoven, The Netherlands; jurgen.piek@catharinaziekenhuis.nl; 6 Department of Obstetrics and Gynecology, Medisch Spectrum Twente, 7512 KZ Enschede, The Netherlands; n.reesink-peters@mst.nl; 7 Department of Public Health, Erasmus MC, University Medical Centre Rotterdam, 3015 GD Rotterdam, The Netherlands; s.polinder@erasmusmc.nl

**Keywords:** advanced stage ovarian cancer, quality-of-life, cytoreductive surgery, PlasmaJet

## Abstract

**Simple Summary:**

It is important to take into account a patient’s quality-of-life after surgery, especially after introducing a new surgical device. The aim of this study was to determine whether the use of the PlasmaJet Surgical device during surgery has an effect on the quality-of-life of patients with advanced ovarian cancer. This study showed that the use of the PlasmaJet Surgical device during surgery leads to a higher quality-of-life than surgery with electrocoagulation alone. The difference in quality-of-life between the groups is mainly in physical and role functioning, fatigue, and pain. A possible explanation could be the differences in tissue damage when working with different equipment during surgery. The PlasmaJet infiltrates the tissue less deeply than electrosurgery. Especially in surgery involving extensive peritoneal stripping, the surgeon must be aware of the effect of the instrument used.

**Abstract:**

Background: Knowledge of quality-of-life after cytoreductive surgery is important to counsel patients with advanced-stage epithelial ovarian cancer prior to surgery. The aim of this study was to determine whether the use of the PlasmaJet Surgical device during cytoreductive surgery has an effect on the quality-of-life of patients with advanced epithelial ovarian cancer. Methods: Data included in this prospective observational study were derived from the PlaComOv study, in which patients with advanced epithelial ovarian cancer were randomly assigned to have cytoreductive surgery with or without adjuvant use of the PlasmaJet. Quality-of-life was measured before surgery and one, six, 12, and 24 months after surgery with three questionnaires: the EORTC QLQ-C30, QLQ-OV28, and EQ-5D-5L. Results: Between 2018 and 2020, 326 patients were enrolled in the trial. The overall response rate was high, with the lowest response rate at 24 months of 77%. At 6 months, quality-of-life was higher in the intervention group (95%CI 0.009; 0.081, *p* = 0.045). At 12 months, quality-of-life was higher in the intervention group with fewer symptoms of fatigue, appetite loss, and diarrhea (95%CI 0.6; 10,0, *p* = 0.027); similarly, patients in the intervention group reported a better body image (95%CI −14.2; −3.0, *p* = 0.003) and a higher score on the visual analog scale (95%CI 1.99; 11.15, *p* = 0.005). At 24 months postoperatively, no further difference was found between the two groups except for pain (95%CI −12.9; −0.8, *p* = 0.027) and body image (95%CI −13.808; −0.733, *p* = 0.029). A higher quality-of-life in the intervention group was partially explained by the mediator ‘surgery outcome’. Conclusions: This study demonstrated knowledge of patients’ quality-of-life until two years after cytoreductive surgery. The use of the PlasmaJet Surgical device during cytoreductive surgery leads to a higher quality-of-life than conventional surgery with electrocoagulation alone. Even after adjustment for the mediator of surgical outcome, a higher quality-of-life was seen in patients who had surgery with the use of the PlasmaJet device.

## 1. Introduction

While cytoreductive surgery (CRS) is considered an effective method for treating patients with advanced-stage epithelial ovarian cancer (EOC), CRS is a complex treatment that may have a considerable impact on a patient’s quality-of-life (QoL) after surgery [1,2,3]. Patients are selected for the procedure using quantitative prognostic indicators, such as imaging findings, CA125 level, response to neoadjuvant chemotherapy, and comorbidity [4,5,6,7,8,9,10,11]. To fully inform patients before CRS in the process of shared decision-making, knowledge of the effect of QoL after CRS is required [12,13,14]. In particular, QoL after surgery may be a decisive factor in choosing to perform CRS with or without a particular device, such as the PlasmaJet, which has been found to increase the percentage of complete CRS without an increase in complications [15]. The PlasmaJet is a thermal plasma energy device. An electrical current is discharged across the device elements inside, where argon gas is heated to generate plasma.

Studies related to QoL in women who had undergone CRS with the addition of bevacizumab or hyperthermic intraperitoneal chemotherapy (HIPEC) showed that the surgery did not adversely impact QoL [16,17,18]. Additionally, no clinical difference in QoL was found between patients who underwent primary CRS and patients who underwent interval CRS [19]. Furthermore, a recent surgery-related study found no difference in QoL between patients who had undergone surgery of lower complexity and those who had undergone extensive surgery [1,2,3]. All studies had a follow-up between 12 and 16 months postoperatively.

While these studies indicated that CRS as such did not affect patients’ QoL, so far, no study has been published that compared the QoL of patients who underwent a conventional CRS and a CRS with the use of the PlasmaJet Surgical device [20]. The PlasmaJet emits a high-energy jet of argon plasma for direct tissue effects and is able to cut or vaporize small tumor foci [21].

The aim of this study was to determine whether the use of the PlasmaJet Surgical device during CRS leads to a higher QoL than surgery without the PlasmaJet. The primary research question was whether a difference in QoL was seen in women undergoing CRS with or without the PlasmaJet Surgical device at 12 and 24 months postoperatively. Secondary outcomes were the effects of the mediators ‘surgical outcome’ and ‘having of a colostomy’ on QoL.

## 2. Materials and Methods

### 2.1. Study Population

Data included in this study were derived from the PlaComOv study, a single-blinded multicenter randomized controlled trial [22]. In thirteen cancer centers in the Netherlands, patients with the International Federation of Gynecology and Obstetrics (FIGO) stage IIIB-IV ovarian cancer were included in the study who were suitable to receive standard treatment, which consists of CRS and chemotherapy [23]. Those patients were randomized to CRS with or to CRS without the adjuvant use of the PlasmaJet device [15]. The PlaComOv study evaluated the effectiveness of the PlasmaJet surgical device in the treatment of advanced EOC. The study was approved by the Medical Ethics Review Board of the Erasmus University Medical Center, Rotterdam, the Netherlands (NL62035.078.17).

### 2.2. Quality-of-Life Assessment

QoL was assessed preoperatively and one, six, 12, and 24 months postoperatively [22]. Patients had the choice to receive the questionnaires digitally or by post. If the questionnaires were not fully completed after 1 week, an automatic reminder was sent. The online questionnaires were sent by GEMS Tracker (GEneric Medical Survey Tracker, Erasmus MC, Rotterdam, The Netherlands), a software package for the distribution of questionnaires and forms during clinical research and quality registrations in healthcare. GEMS Tracker is developed at the Erasmus MC, The Netherlands, in collaboration with several partners. The software is published under an open-source license (new BSD).

Quality-of-life was measured by three validated questionnaires: the QLQ-C30 and QLQ-OV28 of the European Organization for Research and Treatment of Cancer (EORTC, https://qol.eortc.org/, accessed on 28 June 2023) and the EuroQol (EuroQol—EQ-5D) five-dimensional questionnaire (EQ-5D-5L) [24,25,26].

The first questionnaire, the QLQ-C30 (version 3), is a 30-item questionnaire used for patients with cancer [24]. This questionnaire consists of a global health scale, functioning scales (physical, role, emotional, cognitive, and social), and a symptom scale (fatigue, nausea/vomiting, pain, dyspnoea, insomnia, appetite loss, constipation, and diarrhea). The QLQ-C30 scores were transformed to continuous scales from 0 to 100. Higher scores on the global health scale and the functioning scales indicate a higher level of functioning and a better QoL.

The second questionnaire, the QLQ-OV28, is a 28-item questionnaire designed as a supplement to the QLQ-C30 questionnaire for patients with ovarian cancer [25]. The QLQ-OV28 consists of seven symptom scales associated with ovarian cancer: abdominal/gastrointestinal symptoms, peripheral neuropathy, hormonal, body image, attitude to disease/treatment, chemotherapy side effects, and sexuality.

Higher scores on the symptom scales of both QLQ-C30 and QLQ-OV28 indicate a higher level of symptoms or problems. A change in score of five to ten points on the QLC-C30 and QLQ-OV28 global scale is considered small, a change of ten to 20 points is considered moderate, and a change of more than 20 points is considered large [27,28].

The third questionnaire, the EQ-5D-5L, is a descriptive measurement of health and consists of five dimensions covering mobility, self-care, usual activities, pain/discomfort, and anxiety/depression [26]. Each dimension has five response levels. These response levels express the severity of each dimension: no problems, slight problems, moderate problems, severe problems, and extreme problems. The total score for all dimensions is converted into a health state profile. The EQ-5D-5L questionnaire also includes a visual analog scale (VAS), which provides a quantitative measure (0 to 100 scale) of the patient’s perceptions of their overall health on the day of assessment. The endpoints are labeled between ‘The worst health you can imagine’(0) and ‘The best health you can imagine’(100).

### 2.3. Statistical Analysis

All analyses were performed following the intention-to-treat principle. Patient characteristics and response rates at each follow-up were evaluated using descriptive statistics. Descriptive statistics were also used to graphically present the mean QoL scores over time stratified for the intervention.

For the primary study objective, a generalized estimation equations (GEE) analysis with an independent correlation matrix was performed to determine the effect of CRS with the PlasmaJet compared to the control group on QoL one, six, 12, and 24 months postoperative. Time was added as a categorical variable presented by dummy variables. Interaction terms between intervention and time were added as fixed covariates to assess the difference in QoL per time point. The analysis was adjusted for baseline scores by adding the time-independent baseline QoL variables. Women who were no longer alive at the time of analysis were removed from the analysis. For non-responders, data were imputed.

For the secondary outcomes, a mediation analysis was performed to investigate if surgical outcome and colostomy mediated the effect of CRS with the PlasmaJet on QoL (Figure S1). The direct effect of the PlasmaJet on QoL (c’) was analyzed by adding the mediators as independent fixed variables to the existing GEE model (Figure S1). The effect of the mediators on the QoL (b) at 12 and 24 months was analyzed with a new GEE model with an independent correlation matrix.

Descriptive analyses were performed in IBM SPSS Statistics 28; all other analyses were performed with Rstudio version 3. *p*-values *p* < 0.05 were considered statistically significant.

## 3. Results

From February 2018 to September 2020, a total of 326 patients were included in the PlaComOv study. Table 1 presents the baseline characteristics, which were equally divided between the two groups. The mean age was 65.7 (SD10.4) years. The mean body mass index was 25.3 (SD4.9) kg/m^2^. FIGO stage III disease was present in 226 patients (69%), and FIGO stage IV disease was present in 100 patients (31%). World Health Organization (WHO) performance status was predominantly 0–1 (86%). A primary CRS was performed in 13% of the patients and an interval CRS in 87%. A HIPEC procedure was applied in 19% of the patients.

**Figure 1:**
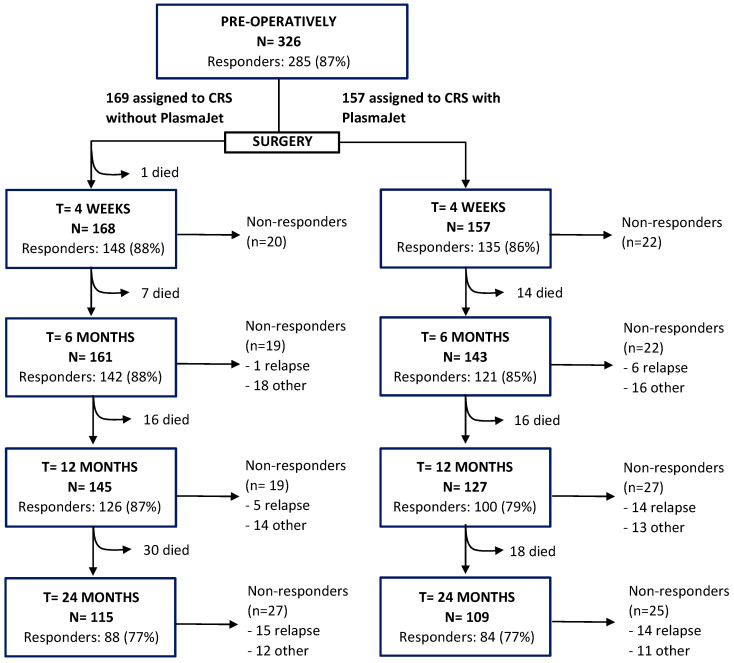
Flow chart of inclusions: responders and non-responders.

Of all patients, 157 patients (48%) were randomized into the intervention group and 169 patients (52%) into the control group (Figure 1). Complete CRS was reached in 76% of the patients in the intervention group and in 68% of the control group (*p* = 0.131). Surgical outcomes are described in Table S1. A colostomy was performed in 11 patients (7%) of the intervention group and in 21 patients (12%) of the control group (*p* = 0.100) (Table S1). Complications are described in Table S2 and did not significantly differ between groups.

### 3.1. Response Rate

About half of the patients (52%) completed the questionnaire digitally, and all other respondents preferred to receive and submit their questionnaire on paper. Figure 1 presents the response rates for completing the three questionnaires. The overall response rate was high, with the lowest percentage of 172 respondents (77%) of all patients surviving 24 months postoperatively. Of all patients who did not respond, 17%, 41%, and 56% had a recurrence at, respectively, six, 12, and 24 months postoperatively.

### 3.2. EORTC QLQ-C30

**Figure 2:**
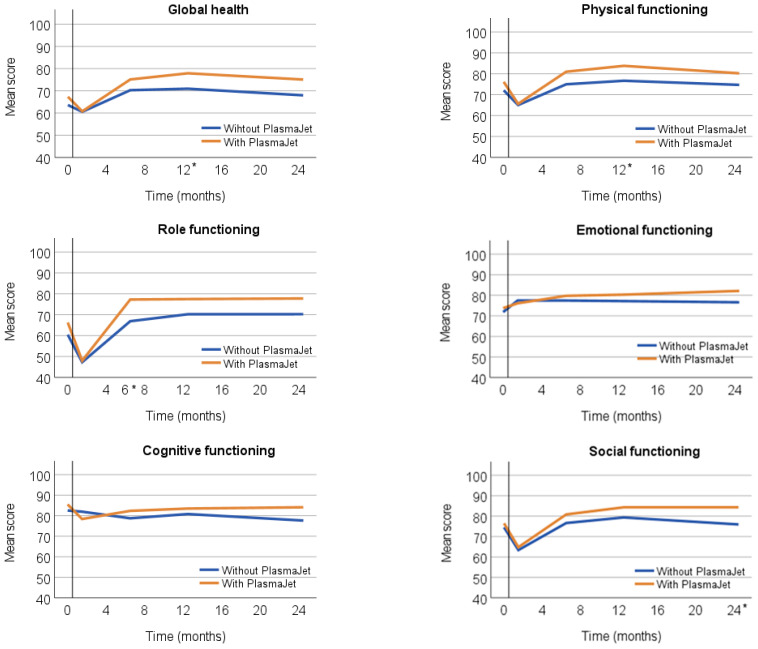
Outcome EORTC QLQ-C30 for every domain at five different time points (preoperative and 1, 6, 12, and 24 months postoperative) for patients who had surgery with or without the PlasmaJet. * *p* < 0.05.

At 12 months postoperatively, the mean score for global health in the EORTC QLQ-C30 in the intervention group was 77.9, and in the control group, 71.0 (Figure 2). Adjustment for the baseline score in both groups revealed a difference of 5.3 points (95%CI 0.60; 10.01, *p* = 0.027). At 24 months postoperatively, the mean score for global health in the intervention group was 75.1 and in the control group 68.0, with a difference of 4.8 points (*p* = 0.083) (Table 2).

Postoperatively, all functioning scales in the EORTC QLQ-C30 showed higher scores in the intervention group than in the control group, which were significantly higher for physical functioning (4.7 points at 12 months, 95% CI 0.12; 9.18, *p* = 0.044), role functioning (7.8 points at 6 months, 95% CI 1.6; 13.9, *p* = 0.014), and social functioning (7.7 points at 24 months, 95% CI 0.5; 14.8, *p* = 0.048) (Table S3). During all the time points, equal QoL scores were found between the groups with regard to cognitive and emotional functioning (Figure 2).

Postoperatively, the symptom scales in the EORTC QLQ-C30 showed fewer symptoms in the intervention group with regard to symptoms of fatigue (−8.0 points at 12 months, 95% CI −13.2; −2.9, *p* = 0.002), appetite loss (−6.1 points, 95% CI −11.4; −0.8, *p* = 0.024,) and diarrhea (−5.2 points, 95% CI −10.0; −0.5, *p* = 0.031). At 24 months postoperatively, the EORTC QLQ-C30 showed fewer symptoms of pain in the intervention group (−6.8 points, 95% CI −12.9; −0.8, *p* = 0.027) (Table S3).

### 3.3. EORTC QLQ-OV28

**Figure 3:**
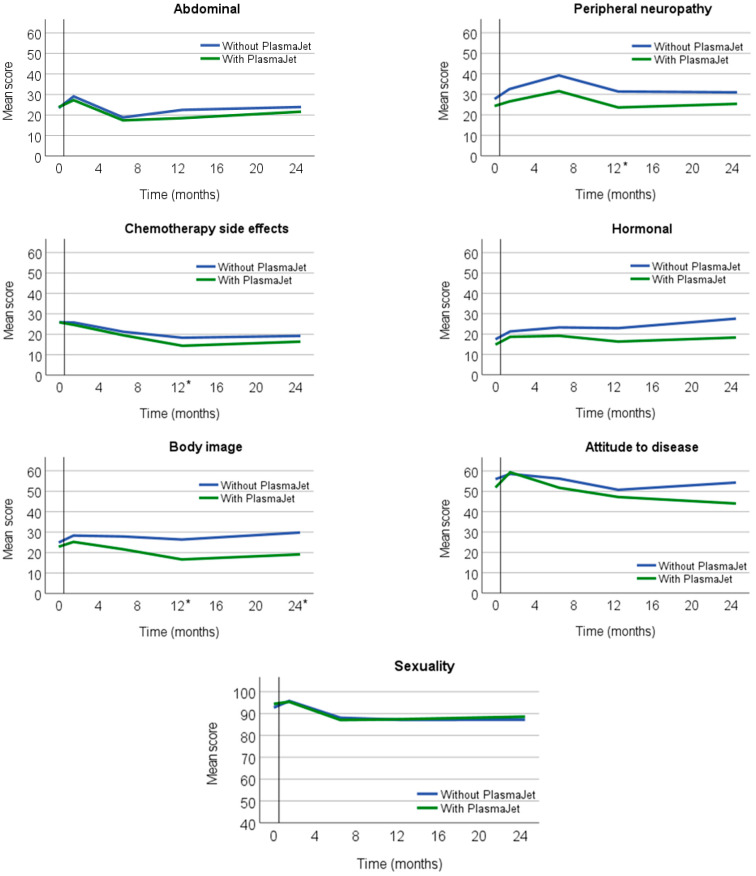
Outcome EORTC QLQ-OV28 for every domain at five different time points for patients who had surgery with or without the PlasmaJet. * *p* < 0.05.

At 12 and 24 months postoperatively, the symptom scores in the EORTC QLQ-OV28 showed a better score in the intervention group for body image than in the control group (−8.6 points, 95% CI −14.3; −3.0, *p* = 0.003) (Table 2). The differences in abdominal symptoms and attitude to disease were −3.9 points (*p* = 0.074) and −5.7 points (*p* = 0.091), respectively (Figure 3).

### 3.4. EQ-5D-5L

At six months postoperatively, the EQ health status demonstrated a higher score for the intervention group (0.80 vs. 0.76, 95% CI 0.009; 0.081, *p* = 0.045) (Table 2). At 12 months postoperatively, the EQ VAS showed a higher score for the intervention group (75.8 versus 68.3 points (95% CI 2.0; 11.2, *p* = 0.005, Figure S2)).

### 3.5. Secondary Outcomes

A mediation analysis was performed to investigate if surgical outcome and colostomy mediated the effect on QoL (Figure S1). Table S4 shows the effect of surgical outcomes on QoL. At 12 months postoperatively, the mean score for global health in the EORTC QLQ-C30 was 77.1 in the group with a complete CRS and 62.8 in the group without a complete CRS (95%CI 5.6; 18.1, *p* = 0.002). At 24 months postoperatively, no difference in the mean score for global health was seen.

Table S5 shows the effect of a colostomy on QoL. No differences in the mean scores for global health in the EORTC QLQ-C30 were seen at 12 or 24 months postoperatively.

Table 2 and Table S3 demonstrate the total and the direct effect of the PlasmaJet on QoL. Because the total effect on QoL is partially explained by the surgical outcome, the direct effect of the PlasmaJet on QoL is lower. However, the direct effect of the PlasmaJet on the QoL remained statistically significant on role functioning, fatigue, diarrhea, body image, and the VAS score.

## 4. Discussion

The aim of this study was to determine whether the use of the PlasmaJet Surgical device during CRS leads to a different QoL than conventional surgery with electrocoagulation only. In our single-blinded randomized PlaComOv study, 326 patients with advanced-stage ovarian cancer were requested to complete QoL questionnaires until two years after CRS.

The response rate in this study was high, and even at 24 months postoperatively, 172 of 224 (77%) patients alive completed the questionnaires. A possible explanation for the high response rate was that patients had the choice to receive the questionnaires digitally or by post, and a reminder was sent if the patients did not complete their questionnaires within one week.

In general, four weeks after CRS, a lower QoL was seen for all patients compared to QoL before surgery (Figure 2 and Figure 3). During follow-up, however, patients indicated a QoL that was equal to or higher than at diagnosis. This finding was in line with previous studies [1,2,3].

At six months postoperatively, the EQ health status was significantly higher in the intervention group. At 12 months postoperatively, the mean global health score was significantly higher in the intervention group than in the control group. Patients in the intervention group had a significantly better body image at 12 and 24 months postoperatively. At 12 months postoperatively, the EQ-VAS was higher in the intervention group.

At 12 months postoperatively, the highest differences in scores between the intervention and the control group were seen for body image, with 8.6 points, and fatigue, with 8.0 points.

The difference in QoL between the groups was clinically small by modern criteria but statistically significant [27,28]. Nevertheless, in patients with a high risk of recurrence of ovarian cancer within two years, any improvement in QoL is relevant.

To our knowledge, the finding that QoL is affected by the use of a medical instrument during surgery for patients with advanced EOC has never been described.

Studies related to QoL in women who had undergone CRS with the addition of bevacizumab or hyperthermic intraperitoneal chemotherapy (HIPEC) showed that the surgery did not adversely impact QoL [16,17,18]. Additionally, no clinical difference in QoL was found between patients who underwent primary CRS and patients who underwent interval CRS [19]. Furthermore, a surgery-related study found no difference in QoL between patients who had undergone surgery of lower complexity and those who had undergone extensive surgery [1,2,3]. All studies had a follow-up between 12 and 16 months postoperatively.

The mediation analysis showed that the direct effect of the PlasmaJet on the QoL was less pronounced after correction for the mediated effect of higher rates of CRS and fewer colostomies compared to the total effect. This suggests that the mediators could partially explain the effect of the PlasmaJet on the QoL.

In a subset analysis to analyze the effect of a colostomy on QoL, no significant differences in the mean scores for global health were seen between patients with or without a colostomy. However, the number of colostomies in this study was small. In addition, not every patient with a colostomy completed the questionnaires. As a result, we could not comment on the effect of a colostomy on QoL.

No sensitivity analysis was performed for all patients who underwent surgery with the use of the PlasmaJet. Patients with less extensive disease and in whom the PlasmaJet was not used would be transferred to the control group. In this case, more patients in the control group would have less extensive disease, and the QoL would automatically be higher in this group.

It is notable that the difference in QoL between the groups is mainly in physical and role functioning, fatigue, and pain. A possible explanation could be the differences in tissue damage when working with different equipment during surgery. The PlasmaJet infiltrates the tissue less deeply than electrosurgery [29]. With less tissue damage, the process of tissue repair (inflammation, proliferation by fibrogenesis, and angiogenesis and remodeling) will proceed differently than when there is more tissue damage. Especially in surgery involving extensive peritoneal stripping, the surgeon must be aware of the effect of the instrument used.

CRS with the PlasmaJet improved various QoL outcomes. At 12 months postoperatively, the adjuvant use of the PlasmaJet for advanced EOC resulted in a higher QoL, which was partially explained by the mediator ‘surgical outcome’. This means that the use of the PlasmaJet can be considered despite a higher cost per procedure [30].

Further research should specifically be focused on the difference in QoL of patients who will undergo CRS and those who will decline surgery. Knowledge of patients’ QoL of both groups, combined with survival data, is needed to fully inform patients in the process of shared decision-making. Thereafter, patients can make an informed decision to undergo or refuse a CRS.

Obtaining more data on the effect of a colostomy on QoL in patients with advanced EOC may be possible if a specific instrument about stoma care is used. More information about QoL in patients with a colostomy after CRS could help the surgical team in perioperative decision-making.

### Strengths and Limitations

This study demonstrated data up to 24 months postoperatively in contrast to previous studies that described QoL up to a maximum of 16 months postoperatively. The overall response rate was high, with the lowest response rate at 24 months postoperatively: 172 of 224 patients alive (77%) completed the questionnaires. The number of non-responders was the same in both groups. A logical dropout rate was because patients died of the disease. In contrast to other studies and because of the prospective study design, the percentage of the non-responders who had a relapse was known (Figure 1).

Our study used an additional short validated questionnaire, the EQ-5D-5L, in contrast to other studies among QoL of patients with advanced EOC [1,16,17,18].

A limitation of this study is that it was impossible to apply a correction for the QoL of the non-respondents. Previously, Stark et al. made a correction for the non-responders who had a recurrence of disease [18]. We waived this because there is insufficient scientific evidence to fill in a fictitious value for QoL. A proportion of our non-responders had a relapse, and it is plausible that they would have a lower QoL than the median QoL. On the other hand, the non-responders without disease and in relatively good health could have had a better QoL, improving the overall outcome in QoL.

## 5. Conclusions

This study demonstrated knowledge of patients’ QoL until two years after CRS to fully inform patients preoperatively. Even after adjustment for the mediator of surgical outcome, a higher QoL was seen in patients who had surgery with the use of the PlasmaJet device at 12 months postoperatively.

## Figures and Tables

**Table 1:** Patient characteristics.

	Total n = 326 (%)	PlasmaJet n = 157 (%)	Control n = 169 (%)
**Age (years)**			
Mean [SD]	65.7 [10.4]	66.1 [9.6]	65.3 [11.2]
Median [min, max]	66.7 [20.4, 86.1]	67.5 [28.9, 81.3]	66.2 [20.4, 86.1]
**BMI**			
Mean [SD]	25.3 [4.9]	24.8 [5.3]	25.7 [4.4]
Median [min, max]	24.6 [17.2, 57.1]	24.0 [17.2, 57.1]	24.9 [17.3, 40.7]
**FIGO stage**			
IIIB	22 (6.7)	11 (7.0)	11 (6.5)
IIIC	204 (62.6)	96 (61.1)	108 (63.9)
IV	100 (30.7)	50 (31.8)	50 (29.6)
**WHO-performance status**			
0	171 (52.8)	82 (52.2)	89 (53.3)
1	109 (33.6)	56 (35.7)	53 (31.7)
2	17 (5.2)	9 (5.7)	8 (4.8)
3	7 (2.2)	2 (1.3)	5 (3.0)
4	1 (0.3)	1 (0.6)	0 (0.0)
**Surgery**			
Primary CRS	44 (13.5)	20 (12.7)	24 (14.2)
Interval CRS	282 (86.5)	137 (87.3)	145 (85.8)
HIPEC	61 (18.7)	29 (18.5)	32 (18.9)

**Table 2:** Mean scores of the EORTC QLQ-C30, QLQ-OV28, and EQ-5D-5L at 12 and 24 months after surgery for patients who underwent surgery with or without the PlasmaJet.

	Mean Score		Total Effect (95% CI)	*p*-Value	Direct Effect (95% CI)	*p*-Value
	PlasmaJet	Control				
**EORTC QLQ-C30**						
**Global health**						
12 months	77.9	71.0	5.3 (0.6; 10.0)	0.027 *	4.4 (−0.3; 9.0)	0.064
24 months	75.1	68.0	4.8 (−0.6; 10.3)	0.083	3.9 (−1.5; 9.3)	0.160
**EORTC QLQ-OV28**						
**Abdominal**						
12 months	18.5	22.5	−3.9 (−8.2; 0.4)	0.074	−3.6 (−7.9; 0.7)	0.099
24 months	21.6	23.9	−1.1 (−5.7; 3.5)	0.631	−0.8 (−5.5; 3.9)	0.728
**Peripheral neuropathy**						
12 months	23.6	31.4	−5.9 (−11.7; −0.2)	0.043 *	−5.3 (−11.0; 0.5)	0.076
24 months	25.4	31.0	−3.0 (−9.1; 3.0)	0.328	−2.3 (−8.5; 3.9)	0.462
**Chemo side effects**						
12 months	14.4	18.3	−3.6 (−6.9; −0.3)	0.032 *	−3.3 (−6.7; −0.01)	0.049 *
24 months	16.4	19.2	−2.4 (−6.7; 2.0)	0.292	−2.081 (−6.5; 2.4)	0.359
**Hormonal**						
12 months	16.3	22.9	−5.5 (−11.3; 0.2)	0.060	−5.6 (−11.4; 0.2)	0.058
24 months	18.3	27.6	−4.6 (−11.8; 2.7)	0.215	−4.6 (−11.9; 2.6)	0.211
**Body image**						
12 months	16.7	26.4	−8.6 (−14.2; −3.0)	0.003 *	−8.4 (−14.1; −2.7)	0.004 *
24 months	19.1	39.8	−7.3 (−13.8; −0.7)	0.029 *	−7.1 (−13.6; −0.5)	0.036 *
**Attitude to disease**						
12 months	47.2	50.8	−1.4 (−7.5; 4.6)	0.645	−0.5 (−6.5; 5.5)	0.863
24 months	44.0	54.3	−5.7 (−12.2; 0.9)	0.091	−4.7 (−11.3; 1.8)	0.158
**Sexuality**						
12 months	87.4	87.1	−0.7 (−5.0; 3.7)	0.759	−0.5 (−4.8; 3.9)	0.830
24 months	88.6	87.2	−0.8 (−5.2; 3.6)	0.717	−0.6 (−4.9; 3.8)	0.791
**EQ-5D-5L**						
**Health status**						
12 months	0.80	0.76	0.021 (−0.029; 0.071)	0.402	0.012 (−0.037; 0.061)	0.439
24 months	0.80	0.77	0.011 (−0.043; 0.066)	0.678	0.002 (−0.052; 0.056)	0.890
**VAS**						
12 months	75.8	68.3	6.6 (2.0; 11.2)	0.005 *	5.8 (1.2; 10.4)	0.014 *
24 months	72.0	68.9	2.4 (−3.0; 7.8)	0.385	1.563 (−3.8; 6.9)	0.569

Total effect: score corrected for preoperative score. Direct effect: score corrected for preoperative score and effect of the mediators ‘Surgical outcome’ and ‘Colostomy’. * = *p* < 0.05.

## Data Availability

Research data at Erasmus MC is generated, stored, and made accessible in accordance with legal, academic, and ethical requirements. This study and all persons involved have knowledge of and comply with the most recent version of the Erasmus MC Research Code, which complies with all current laws and regulations. Data will be handled by practicing the FAIR principles (Findable, Accessible, Interoperable, and Reusable) according to the Handbook for Adequate Natural Data Stewardship (HANDS) developed by the Federation of Dutch UMCs. All research data is handled confidentially in accordance with legislation and conditions imposed by The Dutch Data Protection Authority. The research data from this study is stored in a long-term archive on secured network servers, with regular backup and limited access. In accordance with the Netherlands Code of Conduct for Scientific Practice, raw data is stored for a period of at least ten years. Permission for third persons to access the data will only be granted by the PI on certain conditions.

## Supplementary Materials

The following supporting information can be downloaded at: https://www.mdpi.com/article/10.3390/cancers15153947/s1, Figure S1: Mediation analysis; Figure S2: Outcome EQ-5D-5L for patients who underwent surgery with or without the PlasmaJet; Table S1: Surgical outcome; Table S2: Surgical complications within 30 days; Table S3: Mean scores of the EORTC QLQ-C30, QLQ-OV28, and EQ-5D-5L for every domain at five different time-points for patients who had surgery with or without the PlasmaJet; Table S4: Outcome of the EORTC QLQ-C30 of patients with complete cytoreductive surgery; Table S5: Outcome of the EORTC QLQ-C30 of patients who received a colostomy.

## Abbreviations

CRS	Cytoreductive surgery
EOC	Epithelial Ovarian Cancer
EORTC	European Organization for Research and Treatment of Cancer
FIGO	International Federation of Gynecology and Obstetrics
HIPEC	Hyperthermic intraperitoneal chemotherapy
PlaComOv	Acronym: Will the use of the PlasmaJet surgical device the percentage of Complete cytoreductive surgery for patients with Ovarian cancer
QoL	Quality-of-life
WHO	World Health Organization

## References

[1] Sundar S., Cummins C., Kumar S., Long J., Arora V., Balega J., Broadhead T., Duncan T., Edmondson R., Fotopoulou C. (2022). Quality-of-life from cytoreductive surgery in advanced ovarian cancer: Investigating the association between disease burden and surgical complexity in the international, prospective, SOCQER-2 cohort study. BJOG.

[2] Soo Hoo S., Marriott N., Houlton A., Nevin J., Balega J., Singh K., Yap J., Sethuram R., Elattar A., Luesley D. (2015). Patient-Reported Outcomes After Extensive (Ultraradical) Surgery for Ovarian Cancer: Results From a Prospective Longitudinal Feasibility Study. Int. J. Gynecol. Cancer.

[3] Angioli R., Plotti F., Aloisi A., Capriglione S., Terranova C., Ricciardi R., Montera R., Zullo M.A., Rasi V., Benedetti-Panici P. (2013). Does extensive upper abdomen surgery during primary cytoreduction impact on long-term quality-of-life?. Int. J. Gynecol. Cancer.

[4] Eisenkop S.M., Friedman R.L., Wang H.J. (1998). Complete cytoreductive surgery is feasible and maximizes survival in patients with advanced epithelial ovarian cancer: A prospective study. Gynecol. Oncol..

[5] Tangjitgamol S., Manusirivithaya S., Laopaiboon M., Lumbiganon P., Bryant A. (2016). Interval debulking surgery for advanced epithelial ovarian cancer. Cochrane Database Syst. Rev..

[6] Bristow R.E., Tomacruz R.S., Armstrong D.K., Trimble E.L., Montz F.J. (2002). Survival effect of maximal cytoreductive surgery for advanced ovarian carcinoma during the platinum era: A meta-analysis. J. Clin. Oncol..

[7] Forstner R., Sala E., Kinkel K., Spencer J.A., European Society of Urogenital R. (2010). ESUR guidelines: Ovarian cancer staging and follow-up. Eur. Radiol..

[8] Jayson G.C., Kohn E.C., Kitchener H.C., Ledermann J.A. (2014). Ovarian cancer. Lancet.

[9] Kengsakul M., Nieuwenhuyzen-de Boer G.M., Udomkarnjananun S., Kerr S.J., van Doorn H.C., van Beekhuizen H.J. (2022). Factors Predicting 30-Day Grade IIIa-V Clavien-Dindo Classification Complications and Delayed Chemotherapy Initiation after Cytoreductive Surgery for Advanced-Stage Ovarian Cancer: A Prospective Cohort Study. Cancers.

[10] Kengsakul M., Nieuwenhuyzen-de Boer G.M., Udomkarnjananun S., Kerr S.J., Niehot C.D., van Beekhuizen H.J. (2022). Factors predicting postoperative morbidity after cytoreductive surgery for ovarian cancer: A systematic review and meta-analysis. J. Gynecol. Oncol..

[11] Fotopoulou C., Planchamp F., Aytulu T., Chiva L., Cina A., Ergonul O., Fagotti A., Haidopoulos D., Hasenburg A., Hughes C. (2021). European Society of Gynaecological Oncology guidelines for the peri-operative management of advanced ovarian cancer patients undergoing debulking surgery. Int. J. Gynecol. Cancer.

[12] Stiggelbout A.M., Van der Weijden T., De Wit M.P., Frosch D., Legare F., Montori V.M., Trevena L., Elwyn G. (2012). Shared decision making: Really putting patients at the centre of healthcare. BMJ.

[13] Stiggelbout A.M., Pieterse A.H., De Haes J.C. (2015). Shared decision making: Concepts, evidence, and practice. Patient Educ. Couns..

[14] Resnicow K., Catley D., Goggin K., Hawley S., Williams G.C. (2022). Shared Decision Making in Health Care: Theoretical Perspectives for Why It Works and For Whom. Med. Decis. Mak..

[15] Nieuwenhuyzen-de Boer G.M., Hofhuis W., Reesink-Peters N., Willemsen S., Boere I.A., Schoots I.G., Piek J.M.J., Hofman L.N., Beltman J.J., van Driel W.J. (2022). Adjuvant Use of PlasmaJet Device During Cytoreductive Surgery for Advanced-Stage Ovarian Cancer: Results of the PlaComOv-study, a Randomized Controlled Trial in The Netherlands. Ann. Surg. Oncol..

[16] Koole S.N., Kieffer J.M., Sikorska K., Schagen van Leeuwen J.H., Schreuder H.W.R., Hermans R.H., de Hingh I.H., van der Velden J., Arts H.J., van Ham M. (2021). Health-related quality-of-life after interval cytoreductive surgery with or without hyperthermic intraperitoneal chemotherapy (HIPEC) in patients with stage III ovarian cancer. Eur. J. Surg. Oncol..

[17] Kim J.H., Lee D.E., Lee Y., Ha H.I., Chang Y.J., Chang S.J., Park S.Y., Lim M.C. (2022). Quality-of-life outcomes from the randomized trial of hyperthermic intraperitoneal chemotherapy following cytoreductive surgery for primary ovarian cancer (KOV-HIPEC-01). J. Gynecol. Oncol..

[18] Stark D., Nankivell M., Pujade-Lauraine E., Kristensen G., Elit L., Stockler M., Hilpert F., Cervantes A., Brown J., Lanceley A. (2013). Standard chemotherapy with or without bevacizumab in advanced ovarian cancer: Quality-of-life outcomes from the International Collaboration on Ovarian Neoplasms (ICON7) phase 3 randomised trial. Lancet Oncol..

[19] Kumar S., Long J., Kehoe S., Sundar S., Cummins C. (2019). Quality-of-life outcomes following surgery for advanced ovarian cancer: A systematic review and meta-analysis. Int. J. Gynecol. Cancer.

[20] Nieuwenhuyzen-de Boer G.M., van der Kooy J., van Beekhuizen H.J. (2019). Effectiveness and safety of the PlasmaJet® Device in advanced stage ovarian carcinoma: A systematic review. J. Ovarian Res..

[21] Bourdel N., Chauvet P., Roman H., Pereira B., Somcutian O., Dechelotte P.J., Canis M. (2017). Comparison between resection, bipolar coagulation and Plasmajet(R): A preliminary animal study. Eur. J. Obstet. Gynecol. Reprod. Biol..

[22] Nieuwenhuyzen-de Boer G.M., Hofhuis W., Reesink-Peters N., Ewing-Graham P.C., Schoots I.G., Beltman J.J., Piek J.M.J., Baalbergen A., Kooi G.S., van Haaften A. (2019). Evaluation of effectiveness of the PlasmaJet surgical device in the treatment of advanced stage ovarian cancer (PlaComOv-study): Study protocol of a randomized controlled trial in the Netherlands. BMC Cancer.

[23] Armstrong D.K., Alvarez R.D., Bakkum-Gamez J.N., Barroilhet L., Behbakht K., Berchuck A., Chen L.M., Cristea M., DeRosa M., Eisenhauer E.L. (2021). Ovarian Cancer, Version 2.2020, NCCN Clinical Practice Guidelines in Oncology. J. Natl. Compr. Cancer Netw..

[24] Aaronson N.K., Ahmedzai S., Bergman B., Bullinger M., Cull A., Duez N.J., Filiberti A., Flechtner H., Fleishman S.B., de Haes J.C. (1993). The European Organization for Research and Treatment of Cancer QLQ-C30: A quality-of-life instrument for use in international clinical trials in oncology. J. Natl. Cancer Inst..

[25] Greimel E., Bottomley A., Cull A., Waldenstrom A.C., Arraras J., Chauvenet L., Holzner B., Kuljanic K., Lebrec J., D’Haese S. (2003). An international field study of the reliability and validity of a disease-specific questionnaire module (the QLQ-OV28) in assessing the quality-of-life of patients with ovarian cancer. Eur. J. Cancer.

[26] Herdman M., Gudex C., Lloyd A., Janssen M., Kind P., Parkin D., Bonsel G., Badia X. (2011). Development and preliminary testing of the new five-level version of EQ-5D (EQ-5D-5L). Qual. Life Res..

[27] Osoba D., Rodrigues G., Myles J., Zee B., Pater J. (1998). Interpreting the significance of changes in health-related quality-of-life scores. J. Clin. Oncol..

[28] Cocks K., King M.T., Velikova G., Martyn St-James M., Fayers P.M., Brown J.M. (2011). Evidence-based guidelines for determination of sample size and interpretation of the European Organisation for the Research and Treatment of Cancer Quality-of-life Questionnaire Core 30. J. Clin. Oncol..

[29] Nieuwenhuyzen-de Boer G.M., van de Berg N.J., Gao X.S., Ewing-Graham P.C., van Beekhuizen H.J. (2022). The effects of neutral argon plasma versus electrocoagulation on tissue in advanced-stage ovarian cancer: A case series. J. Ovarian Res..

[30] Nieuwenhuyzen-de Boer G.M., Geraerds A., van der Linden M.H., van Doorn H.C., Polinder S., van Beekhuizen H.J. (2022). Cost Study of the PlasmaJet Surgical Device Versus Conventional Cytoreductive Surgery in Patients With Advanced-Stage Ovarian Cancer. JCO Clin. Cancer Inform..

