# Supplementary material for: Cytoreductive Surgery with the PlasmaJet Improved Quality-of-Life for Advanced Stage Ovarian Cancer Patients
Source: Cancers (Basel). 2023 Aug 3;15(15):3947. doi: 10.3390/cancers15153947 (PMC10416900; doi:10.3390/cancers15153947)
Supplement: Supplementary file 1 [file cancers-15-03947-s001.zip › cancers-2504590-supplementary.pdf]

Supplementary

Table S1. Surgical outcome

	PLASMAJET N=157 (%)	CONTROL N=169 (%)	P-VALUE
**Surgical outcome**			
**Complete**	119 (75.8)	114 (67.5)	0.001
**Optimal**	12 (7.6)	38 (22.5)	
**Suboptimal**	8 (5.1)	8 (4.7)	
**Unresectable**	18 (11.5)	9 (5.3)	
**Complete cytoreductive surgery YES**	119 (75.8)	114 (67.5)	0.131
**Start of surgery**			
**Primary CRS**	20 (12.7)	24 (14.7)	0.722
**Interval CRS**	137 (87.3)	145 (85.3)	
**Operative time (minutes)**			
**mean [SD]**	236 [126]	222 [110]	0.326
**median [min, max]**	210 [29, 671]	194 [48, 595]	
**Missing**	6 (3.8)	4 (2.4)	
**Abdominal drain**	35 (22.3)	50 (29.4)	0.259
**Blood loss (ml)**			
**mean [SD]**	923 [801]	956 [801]	0.712
**median [min, max]**	700 [0, 4300]	845 [0, 6000]	
**Missing**	4 (2.5)	1 (0.6)	
**Transfusion during surgery**	41 (26.1)	45 (26.5)	0.877
**Colostomy**	11 (7.0)	21 (12.4)	0.100
**Intensive Care postoperative**	34 (21.7)	40 (23.5)	0.785
**Intensive Care (days)**			
**mean [SD]**	1.9 (1.9)	1.6 (0.9)	0.339
**median [min, max]**	1.0 [1, 11]	1.0 [1, 5]	
**Hospitalization (days)**			

**mean [SD]**	8.7 [6.5]	7.9 [6.4]	0.221
**median [min, max]**	6.5 [2, 35]	6.0 [2, 51]	
**missing**	3 (1.9)	0	
**Discharge**			
**Home without nursing care**	100 (63.7)	110 (64.7)	0.955
**Home with nursing care**	34 (21.7)	39 (22.9)	
**Nursing home**	4 (2.5)	3 (1.8)	
**Rehabilitation center**	2 (1.3)	2 (1.2)	
**Hotel providing nursing care**	9 (5.7)	12 (7.1)	
**Hospice**	1 (0.6)	0	
**Death**	0	0	

CRS = Cytoreductive surgery

Table S2 Surgical complications within 30-days

	PLASMAJET n=139 (%)	Control n=158 (%)	P-value
**Bowel laceration post-operative**	2 (1.4)	1 (0.6)	0.597
**Bowel obstruction (ileus)**			
**-Conservative**	11 (7.9)	14 (8.9)	1
**-Surgery**	0	0	
**Surgical site infection**			
**Sepsis**	0	4 (2.5)	0.127
**Intra-abdominal abscess**	1 (0.7)	3 (1.9)	0.627
**Urinary tract infection**	8 (5.8)	7 (4.4)	0.735
**Superficial wound infection**	8 (5.7)	4 (2.5)	0.201
**Relaparotomy****	8 (5.8)	3 (1.9)	0.143
**Medical complication**			
**Cardiac**	6 (4.3)	7 (4.4)	1
**Venous thrombo-embolism**	1 (0.7)	2 (1.3)	1
**Deep venous embolism**	1 (0.7)	2 (1.3)	1
**Pulmonary embolism**	2 (1.4)	3 (1.9)	1
**Pulmonary failure**	0	0	1
**Pneumonia**	2 (1.4)	10 (6.3)	0.072
**Respiratory insufficiency**	7 (5.0)	5 (3.1)	0.551
**Renal failure**	1 (0.7)	1 (0.6)	1
**Ureter laceration**	0	0	1
**Gastric perforation**	1 (0.7)	0	1
**Anastomotic leakage**	1 (0.7)	1 (0.6)	1
**Stroke**	1 (0.7)	0	1

**Delirium**	5 (3.6)	1 (0.6)	0.099
**Death (within 30-days)**	0	1 (0.6)	0.319

*All patients with unresectable disease are excluded from analysis and patient who underwent surgery in the Control group with the use of the PlasmaJet.
** Indications intervention group: anastomotic leakage (1), suspicion of anastomotic leakage (2), to continue and finish the interval debulking surgery (1), gastric perforation (1), pancreatic leakage (1), intra-abdominal bleeding (1), pelvic abscess (1).
Control group: anastomotic leakage (1) and suspicion of anastomotic leakage (2).

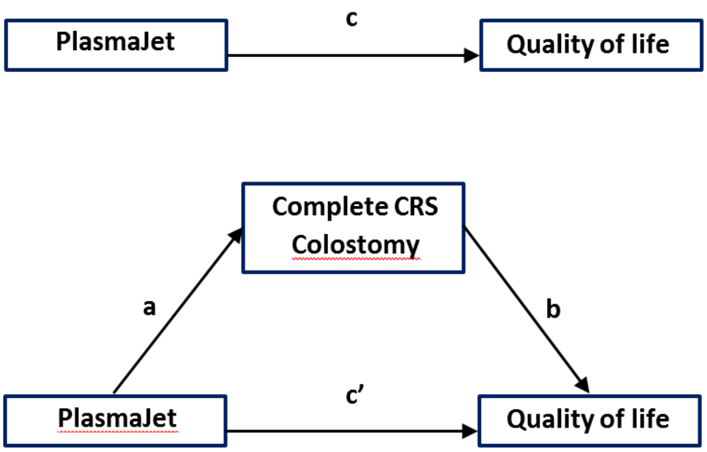

Figure S1 Mediation analysis

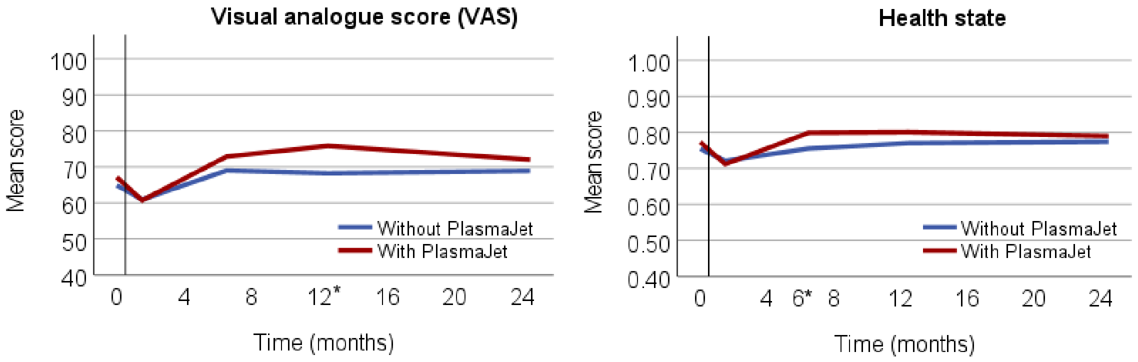

Figure S2 Outcome EQ-5D-5L for patients who had surgery with or without the PlasmaJet. * P < 0.05.

Table S3 Mean scores of the EORTC QLQ-C30, QLQ-OV28 and EQ-5D-5L for every domain at five different time-points (preoperative and 1, 6, 12 and 24 months postoperative) for patients who had surgery with or without the PlasmaJet

	Mean score		Total effect (95%CI)	P-value	Direct effect (95%CI)	P-value
	PlasmaJet	Control				
**EORTC QLQ-C30**						
**Global health**						
Pre-op	67.34	63.63				
4 weeks	60.80	60.59	-0.638 (-4.819;3.543)	0.765	-1.234 (-5.395;2.927)	0.561
6 months	75.14	70.30	3.310 (-1.159;7.780)	0.146	2.591 (-1.839;7.020)	0.251
12 months	77.92	70.97	5.305 (0.595;10.014)	0.027*	4.374 (-0.256;9.004)	0.064
24 months	75.10	67.99	4.828 (-0.627;10.282)	0.083	3.876 (-1.533;9.285)	0.160
**Physical functioning**						
Pre-op	76.16	72.12				
4 weeks	65.63	65.05	-0.692 (-4.867;3.483)	0.745	-1.098 (-5.245;3.049)	0.604
6 months	80.99	74.98	3.689 (-0.185;7.564)	0.062	3.237 (-0.630;7.104)	0.101
12 months	83.80	76.67	4.652 (0.123;9.181)	0.044*	4.115 (-0.338;8.568)	0.070
24 months	80.24	74.70	3.344 (-2.098;8.786)	0.228	2.786 (-2.635;8.207)	0.314
**Role functioning**						
Pre-op	66.30	60.47				
4 weeks	48.02	47.30	0.173 (-6.049;6.395)	0.956	-0.574 (-6.800;5.651)	0.857
6 months	77.27	66.90	7.757 (1.581;13.932)	0.014*	6.880 (0.671;13.089)	0.030*
12 months	77.50	70.24	5.362 (-1.133;11.857)	0.106	4.268 (-2.220;10.756)	0.197
24 months	77.78	70.27	5.800 (-1.523;13.123)	0.120	4.668 (-2.710;12.046)	0.215
**Emotional functioning**						
Pre-op	73.78	71.85				
4 weeks	76.11	77.48	-1.039 (-4.805;2.728)	0.589	-1.324 (-5.045;2.397)	0.485
6 months	79.75	77.47	1.133 (-3.201;5.466)	0.608	0.771 (-3.505;5.047)	0.724
12 months	80.33	77.18	2.378 (-2.401;7.156)	0.329	1.902 (-2.853;6.658)	0.433
24 months	82.14	76.61	2.256 (-2.192;8.703)	0.241	2.767 (-2.649;8.184)	0.316
**Cognitive functioning**						
Pre-op	85.52	82.55				
4 weeks	78.39	81.98	-3.579 (-7.754;0.597)	0.093	-3.561 (-7.744;0.623)	0.095
6 months	82.37	78.76	2.356 (-2.144;6.857)	0.305	2.393 (-2.105;6.891)	0.297
12 months	83.50	80.82	1.631 (-2.927;6.189)	0.483	1.695 (-2.844;6.234)	0.464
24 months	84.13	77.65	4.020 (-1.676;9.716)	0.166	4.085 (-1.630;9.801)	0.161
**Social functioning**						
Pre-op	76.52	74.55				
4 weeks	64.81	63.40	1.678 (-3.697;7.051)	0.541	1.087 (-4.302;6.476)	0.692

6 months	80.85	76.64	3.312 (-2.778;9.402)	0.286	2.604 (-3.459;8.667)	0.400
12 months	84.33	79.36	4.025 (-1.694;9.745)	0.168	3.123 (-2.423;8.669)	0.270
24 months	84.33	75.95	7.662 (0.488;14.836)	0.036*	6.782 (-0.444;13.901)	0.066
**Fatigue**						
Pre-op	34.71	39.94				
4 weeks	47.49	49.32	-0.675 (-5.878;4.527)	0.799	-0.243 (-5.458;4.971)	0.927
6 months	25.99	35.45	-6.458 (-11.588;-1.329)	0.014*	-5.888 (-11.046;-0.729)	0.025*
12 months	24.78	35.54	-8.047 (-13.207;-2.886)	0.002*	-7.265 (-12.409;-2.120)	0.006*
24 month	27.25	36.99	-6.042 (-12.972;0.888)	0.087	-5.234 (-12.206;1.738)	0.141
**Nausea and vomiting**						
Pre-op	9.37	7.32				
4 weeks	15.06	13.29	1.027 (-4.425;6.480)	0.712	1.534 (-3.869;6.983)	0.578
6 months	7.30	4.34	2.918 (-0.867;6.702)	0.131	3.536 (-0.268;7.340)	0.068
12 months	4.83	7.01	-1.823 (-5.613;1.967)	0.346	-1.039 (-4.786;2.709)	0.587
24 months	6.94	9.09	-1.687 (-7.277;3.902)	0.554	-0.876 (-6.489;4.738)	0.760
**Pain**						
Pre-op	21.41	21.85				
4 weeks	30.62	29.05	0.425 (-5.119;5.968)	0.881	1.031 (-4.515;6.578)	0.715
6 months	14.46	17.25	-2.885 (-8.260;2.490)	0.293	-2.142 (-7.523;3.240)	0.435
12 months	15.33	19.97	-5.028 (-11.252;1.195)	0.113	-4.060 (-10.120;2.000)	0.189
24 months	13.10	21.40	-6.846 (-12.932;-0.760)	0.027*	-5.847 (-11.874;0.181)	0.057
**Dyspnoea**						
Pre-op	17.03	20.04				
4 weeks	20.25	20.95	0.468 (-5.084;6.020)	0.869	0.480 (-5.135;6.095)	0.867
6 months	15.43	19.72	-3.052 (-8.756;2.652)	0.294	-3.042 (-8.831;2.747)	0.303
12 months	18.00	20.90	-2.553 (-8.686;3.581)	0.415	-2.546 (-8.790;3.697)	0.424
24 months	18.65	22.73	-2.282 (-9.172;4.608)	0.516	-2.275 (-9.175;4.624)	0.518
**Insomnia**						
Pre-op	28.71	26.58				
4 weeks	31.85	30.85	-2.001 (-7.599;3.597)	0.483	-1.441 (-7.030;4.147)	0.613
6 months	25.62	24.41	-0.071 (-5.779;5.637)	0.981	0.581 (-5.161;6.323)	0.843
12 months	27.67	29.63	-4.003 (-11.817;3.810)	0.315	-3.199 (-11.009;4.611)	0.422
24 months	25.79	27.27	-1.368 (-8.706;5.970)	0.715	-0.541 (-7.850;6.768)	0.885
**Appetite loss**						

Pre-op	19.95	17.12				
4 weeks	27.41	28.83	-2.504 (-9.350;4.341)	0.473	-1.809 (-8.660;5.041)	0.605
6 months	9.92	9.62	-0.592 (-5.948;4.763)	0.828	0.253 (-5.038;5.545)	0.925
12 months	7.33	12.43	-6.085 (-11.385;-0.785)	0.024*	-4.994 (-10.319;0.332)	0.066
24 months	11.11	17.42	-7.141 (-14.406;0.125)	0.054	-6.012 (-13.322;1.299)	0.107
**Constipation**						
Pre-op	17.76	14.86				
4 weeks	24.69	22.97	0.794 (-5.693;7.280)	0.810	0.542 (-5.886;6.970)	0.869
6 months	9.37	11.03	-1.727 (-6.216;2.763)	0.451	-1.893 (-6.363;2.578)	0.407
12 months	11.67	14.81	-3.923 (-9.552;1.706)	0.172	-3.975 (-9.619;1.670)	0.167
24 months	14.28	13.64	0.093 (-5.964;6.149)	0.976	0.033 (-6.003;6.069)	0.991
**Diarrhoea**						
Pre-op	8.03	7.66				
4 weeks	11.36	14.86	-3.139 (-8.719;2.441)	0.270	-2.818 (-8.337;2.701)	0.317
6 months	6.61	4.69	1.776 (-2.127;5.679)	0.372	2.133 (-1.754;6.019)	0.282
12 months	6.33	11.11	-5.249 (-10.012;-0.487)	0.031*	-4.829 (-9.531;-0.127)	0.044*
24 months	5.95	10.98	-5.249 (-10.795;0.296)	0.063	-3.820 (-10.303;0.664)	0.085
**Financial difficulties**						
Pre-op	5.35	8.33				
4 weeks	6.17	6.08	1.045 (-1.898;3.989)	0.486	1.070 (-1.890;4.031)	0.478
6 months	7.16	8.22	0.033 (-3.826;3.891)	0.987	0.084 (-3.832;4.001)	0.966
12 months	8.20	8.00	0.729 (-4.010;5.467)	0.763	0.821 (-3.841;5.483)	0.730
24 months	6.35	9.85	-2.056 (-7.402;3.290)	0.451	-1.960 (-7.248;3.329)	0.467
**EORTC QLQ-OV28**						
**Abdominal**						
Pre-op	23.88	23.45				
4 weeks	27.26	29.09	-2.839 (-6.534;0.856)	0.132	-2.576 (-6.267;1.115)	0.171
6 months	17.42	18.85	-1.774 (-5.370;1.821)	0.333	-1.505 (-5.119;2.108)	0.414
12 months	18.50	22.51	-3.908 (-8.191;0.375)	0.074	-3.622 (-7.926;0.681)	0.099
24 months	21.63	23.92	-1.131 (-5.742;3.480)	0.631	-0.832 (-5.531;3.867)	0.728
**Peripheral neuropathy**						
Pre-op	24.33	27.74				
4 weeks	26.53	32.58	-4.377 (-8.859;0.105)	0.056	-3.896 (-8.370;0.579)	0.088
6 months	31.57	39.24	-5.951 (-11.934;0.032)	0.051	-5.396 (-11.433;0.642)	0.080

12 months	23.61	31.38	-5.930 (-11.684;-0.176)	0.043*	-5.250 (-11.047;0.548)	0.076
24 months	25.38	30.99	-3.032 (-9.114;3.049)	0.328	-2.323 (-8.509;3.865)	0.462
**Chemo side effects**						
Pre-op	25.86	25.92				
4 weeks	24.59	25.75	-1.428 (-4.811;1.955)	0.408	-1.226 (-4.620;2.168)	0.479
6 months	19.52	21.24	-1.697 (-5.327;1.933)	0.359	-1.473 (-5.124;2.178)	0.429
12 months	14.38	18.32	-3.602 (-6.900;-0.303)	0.032*	-3.338 (-6.663;-0.013)	0.049*
24 months	16.40	19.21	-2.355 (-6.743;2.033)	0.292	-2.081 (-6.527;2.365)	0.359
**Hormonal**						
Pre-op	14.84	17.46				
4 weeks	18.66	21.32	-2.567 (-7.076;1.942)	0.264	-2.580 (-7.105;1.946)	0.264
6 months	23.29	19.17	-3.184 (-8.841;2.473)	0.270	-3.215 (-8.922;2.491)	0.269
12 months	16.32	22.93	-5.535 (-11.293;0.224)	0.060	-5.595 (-11.374;0.185)	0.058
24 months	18.31	27.59	-4.579 (-11.820;2.661)	0.215	-4.641 (-11.917;2.634)	0.211
**Body image**						
Pre-op	22.87	24.94				
4 weeks	25.25	28.34	-4.081 (-9.260;1.098)	0.122	-3.879 (-9.051;1.293)	0.141
6 months	27.90	21.67	-4.912 (-10.695;0.871)	0.096	-4.709 (-10.525;1.108)	0.112
12 months	16.67	26.40	-8.613 (-14.248;-2.978)	0.003*	-8.401 (-14.082;-2.721)	0.004*
24 months	19.14	39.81	-7.270 (-13.808;-0.733)	0.029*	-7.052 (-13.633;-0.470)	0.036*
**Attitude to disease**						
Pre-op	51.91	56.01				
4 weeks	59.37	58.58	1.129 (-3.223;5.480)	0.611	1.835 (-2.450;6.121)	0.401
6 months	51.76	56.26	-1.146 (-6.749;4.456)	0.688	-0.376 (-5.937;5.184)	0.894
12 months	47.22	50.76	-1.424 (-7.482;4.634)	0.645	-0.529 (-6.530;5.472)	0.863
24 months	44.03	54.32	-5.653 (-12.214;0.908)	0.091	-4.731 (-11.303;1.841)	0.158
**Sexuality**						
Pre-op	94.40	92.74				
4 weeks	95.40	95.75	-1.175 (-3.791;1.441)	0.379	-1.044 (-3.691;1.603)	0.439
6 months	87.03	88.06	-2.069 (-5.735;1.596)	0.268	-1.910 (-5.592;1.773)	0.309
12 months	87.41	87.13	-0.681 (-5.038;3.675)	0.759	-0.474 (-4.803;3.856)	0.830
24 months	88.58	87.22	-0.804 (-5.158;3.551)	0.717	-0.590 (-4.941;3.762)	0.791
**EQ-5D-5L**						
**Health status**						

Pre-op	0.77	0.75				
4 weeks	0.71	0.72	-0.004 (-0.046;0.038)	0.842	-0.011 (-0.052;0.030)	0.605
6 months	0.80	0.76	0.041 (0.009;0.081)	0.045*	0.034 (-0.007;0.074)	0.105
12 months	0.80	0.77	0.021 (-0.029;0.071)	0.402	0.012 (-0.037;0.061)	0.633
24 months	0.79	0.77	0.011 (-0.043;0.066)	0.678	0.002 (-0.052;0.056)	0.945
**VAS**						
Pre-op						
4 weeks	67.16	64.90	-0.754 (-4.518;3.009)	0.694	-1.253 (-5.009;2.502)	0.513
6 months	72.88	69.01	3.175 (-0.514;6.863)	0.091	2.563 (-1.123;6.248)	0.173
12 months	75.84	68.26	6.571 (1.992;11.150)	0.005*	5.774 (1.177;10.370)	0.014*
24 months	72.03	68.91	2.387 (-3.006;7.781)	0.385	1.563 (-3.815;6.940)	0.569

Total effect: score corrected for preoperative score. Direct effect: score corrected for preoperative score and effect of the mediators ‘Surgical outcome’ and ‘Colostomy’. * = p<0.05

Table S4 Outcome of the EORTC QLQ-C30 of patients with complete cytoreductive surgery

	Mean score			
	Complete CRS (n=233)	Non-complete CRS (n=93)	95%CI	p-value
**EORTC QLQ-C30 Global health**				
12 months	77.1	62.8	11.8 (5.6;18.1)	0.002*
24 months	72.7	65.8	3.7 (-4.3;11.7)	0.368

CRS= cytoreductive surgery

Table S5 Outcome of the EORTC QLQ-C30 of patients who received a colostomy

	Mean score			
	Colostomy (n=32)	No Colostomy (n=294)	95%CI	p-value
**EORTC QLQ-C30 Global health**				
12 months	71.7	74.3	-2.4 (-11.2;6.4)	0.588
24 months	71.1	71.5	-2.4 (-5.6;10.4)	0.557
